# Greenhouse gas emission potential of sewage treatment plants in Himachal Pradesh

**DOI:** 10.1038/s41598-023-36825-7

**Published:** 2023-06-14

**Authors:** Apurva Sharma, Satish Kumar Bhardwaj, R. K. Aggarwal, Ravinder Sharma, Ghanshyam Agrawal

**Affiliations:** Department of Environmental Science, College of Forestry, Dr YS Parmar University of Horticulture and Forestry, Nauni, Solan, India

**Keywords:** Environmental impact, Climate change

## Abstract

In recent times, waste management has emerged as a significant environmental challenge, and sewage is among the major contributors due to the rapidly increasing population. Despite sewage treatment plants (STPs) being the solution for the treatment of sewage, they have been identified as sources of greenhouse gas (GHG) emissions. This study aimed to estimate the contribution of STPs to GHG emissions in the state. This was achieved by visiting the sites, filling scientifically designed questionnaires, sample collection as well as computational methods by Intergovernmental Panel on Climate Change. The assessment of direct and indirect emissions from the STPs revealed that emissions were caused by the activated sludge process, electricity consumption, transportation, and sludge storage. Electricity consumption by STPs was responsible for the highest emissions, accounting for 43% of the total emissions, equivalent to 20,823 tCO_2_ eq. The activated sludge process contributed 31% (14,934 tCO_2_ eq) of the emissions, while storage of sludge in landfills accounted for 24% (11,359 tCO_2_ eq). Additionally, transportation contributed 2% (1121 tCO_2_ eq) of the emissions. In total, the STPs in Himachal Pradesh had the potential to contribute 48,237 tCO_2_ eq GHG emissions annually. Thus, the study suggests process-level modifications in STPs of Himachal Pradesh to mitigate GHG emissions. This research provides insight into the GHG emissions from STPs and highlights the need for their management to reduce environmental impacts.

## Introduction

The rapidly growing population and global industrialization have significantly increased the pressure and have tremendously affected wastewater handling structures^1–3^. This susceptibility of the wastewater industry has also resulted in a rise in greenhouse gas (GHG) emissions, raising concerns about the sector's sustainable development^4,5^. Globally, the inadequate disposal practices of sludge as well use of rudimentary processes for the treatment of wastewater are being considered as a source of GHG emissions^6–9^. Furthermore, together with the operation of sewage treatment plants, the fast growth in wastewater volume is resulting in large GHG emissions from wastewater handling facilities^10^. Global researchers have focused on several stages of wastewater handling structures such as sewerage/effluent transportation, building and operation of wastewater and/or sludge treatment units, and reception or reuse water networks, among others, in the context of GHG emissions^11–13^. It is widely known in the literature that the potential for GHG emissions related with WWTS is mostly connected with carbon dioxide (CO_2_), methane (CH_4_), and nitrous oxide (N_2_O) gases^4,14^. The operation of these facilities results in direct emission of greenhouse gases from biological processes such as CO_2_, N_2_O and CH_4_ as well as indirect emissions resulting from energy consumption, transportation etc. which are responsible for CO_2_ emissions^15–17^. Earlier focus of sewage treatment plants was on obtaining good quality effluents, but now due to changed scenario the sustainability of STPs is being considered to ensure economic feasibility and environmental compatibility.

**Figure 1:**
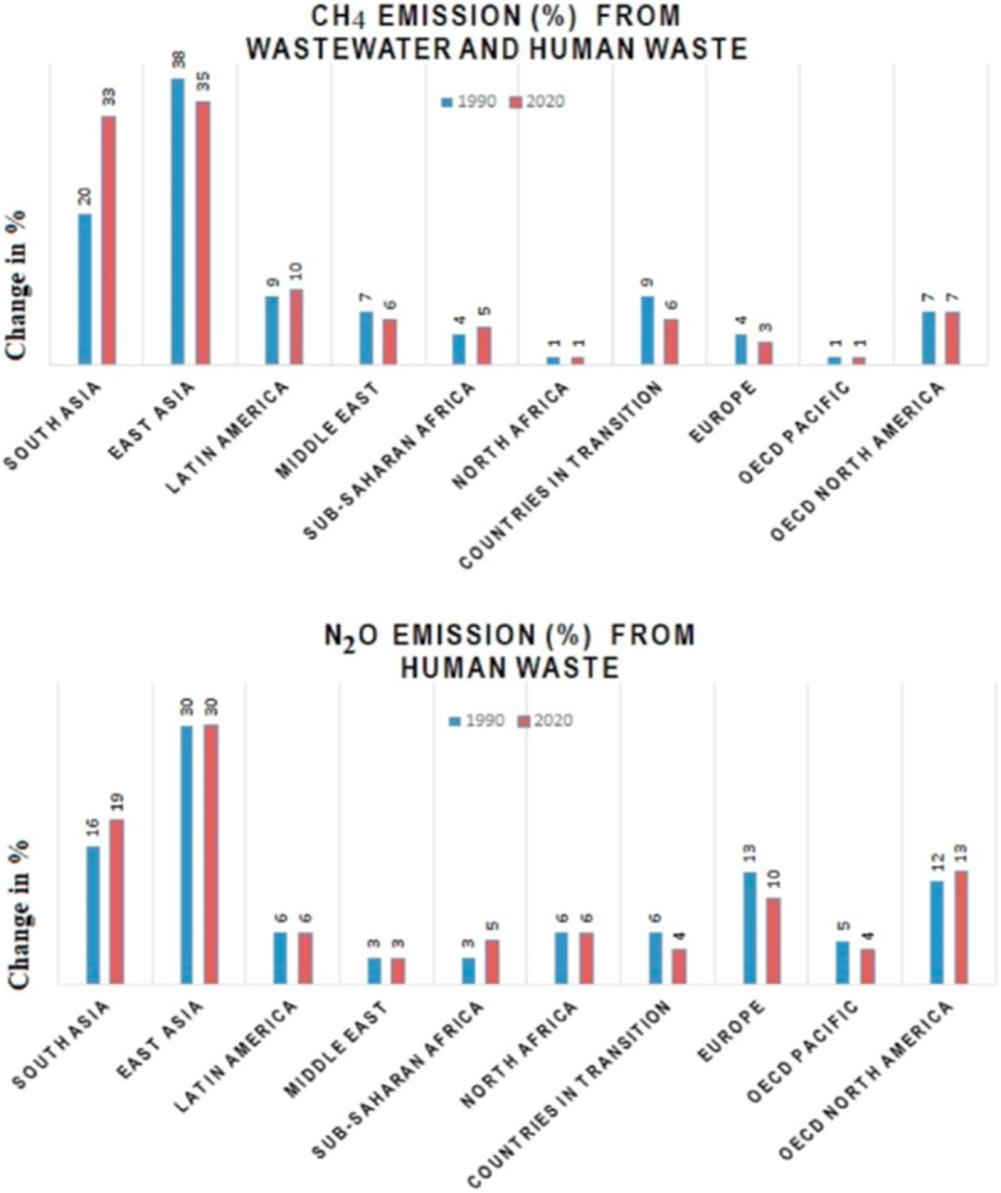
Regional shift in potent emissions from wastewater and human sewage from 1990- 2020^18^.

As seen in Fig. 1^18^, sewage treatment plants and similar structures are potential sources of GHG emissions, with a large proportion of these emissions being reported from developing nations. In the last three decades, CH_4_ emissions from wastewater increased by up to 50% in rapidly developing nations, such as ours (Eastern and Southern Asia)^19,20^. Similarly, worldwide N_2_O estimates though insufficient, primarily based on sewage treatment, increased by 25% in these years. These data clearly indicate the necessity for in-depth research on mitigation strategies for GHG emissions from WWTS. As a result, knowing possible GHG sources and their generating methods is required in order to design a strategic mitigation and/or control plan. Initially, onsite measurement and mathematical modeling approaches were applied to estimate the GHGs emission from WWTS^21^. Later on, advanced approaches such as carbon footprint analysis^22^, life cycle assessment^23^, mass balance analysis^24^, and mechanistic dynamic models^17^ emerged as potentially viable tools and are frequently used for the prediction of the GHGs emission potential of WWTS.

With regard to each GHG source, the N_2_O emitted is generated by nitrification and denitrification processes used to remove nitrogenous compounds from the sewage. Its production occurs mainly in the activated sludge units (90%) while the remaining 10% comes from the grit and sludge storage tanks^25^. The higher COD of wastewater during anaerobic digestion, buffer tank for digestion and storage of dewatered sludge have been reported to be mainly responsible for CH_4_ emission^26^. With respect to CO_2_, its production is attributed to two main factors: biological treatment process, electricity consumption and transportation of sludge. In the main stream of the STP the organic carbon of wastewater is either incorporated into biomass or oxidized to CO_2_. In the sludge line, it is converted mainly to CO_2_ and CH_4_ during anaerobic digestion and finally, methane is oxidized to CO_2_ during biogas combustion.

In Himachal Pradesh, 59,495 million litres of sewage is produced annually and a total of 59 STPs of different capacities are in operation^27^. They are equipped with primary and secondary treatment facilities, which are however, rudimentary and poorly maintained, making them a potential source of GHG emissions. To assess their compatibility with the environment and to fulfil our obligation to reduce these emissions, it is essential to inventorize them from various sources so that their mitigation by making improvements at process level and their proper maintenance may help in making them environmentally compatible.

## Material and methods

### Status of STPs in the state

Sewage treatment plants in the state consist of preliminary treatment, activated sludge process and sludge handling process. The incoming wastewater passes through the screening grills and then into aeration tanks where it is aerated for 5–6 h by electrically operated aerators, then sent to an activated sludge tank. Here sewage is mixed with sludge degrading microbial consortia, stirred continuously, for 4–5 h. It is then sent to settling tanks. Sewage is settled for 4–5 h and the supernatant is separated and released into a nearby waterbody as treated effluent. Settled sludge is pumped onto drying beds and dried sludge is collected in trucks and sent to landfills^28^.

The hilly and slopy terrains with increasing urbanization have unevenly distributed the population in the state. Accordingly, the STPs have been installed in the state by considering the population density. Further, the skewness in the distribution of STPs of different capacities is very high because of this varying degree of urbanization and developmental blocks in the state. In the state there are a total of 59 operational STPs out of which 24 are < 1 MLD, 26 are 1–3 MLD and 9 are > 3 MLD capacity^27^.

### Experimental details

STPs of different capacities in the state were identified and surveyed for their functionality. The three classes discussed above were considered as treatments and seven STPs of each capacity were selected purposefully which were considered as replications. The data was arranged in randomized block design and analysed. Data obtained for a single plant in these categories was used to estimate the total emission from all the plants in the state.

### GHG emission calculations

In order to determine the carbon footprint for STPs in Himachal Pradesh, on-site and off-site GHG emissions were considered. On-site GHG emissions for the plants are mainly generated by activated sludge process. Off-site GHG emissions are generated by energy consumption for STP supplying, sludge transportation and landfilling and degradation of the remaining constituents in the effluent.

There are different methodologies used for determining the carbon footprint of STPs: IPCC-2006, WSAA 2006, LGO-2008, Bridle-2008, NGER-2009^29^ and since no single method was eligible for calculation of process-based footprints, therefore, different methods for all the processes of sewage water treatment were used. Accordingly, standard methods for GHG emissions from electricity consumption^30^; activated sludge process^31^; N_2_O emissions^32^ and sludge transportation^33^ were employed. The data were collected by interviewing the plant managers, visiting the plants and filling scientifically designed questionnaires using field data during 2020–2021. Secondary data necessary for calculations were procured from Jal Shakti Vibhag, Himachal Pradesh.

The calculation of corresponding CO_2eq_ is performed applying the global warming potential (GWP) of 25 kg CO_2eq_/kg CH_4_ and 280 kg CO_2eq_/kg N_2_O referring to a time period of 100 years^34^.

### Off-site GHG emissions

The off-site GHG emission from energy consumption of the sewage treatment system was calculated by energy requirement of plants to operate wastewater and sludge treatment processes and meeting the requirements of administrative buildings and for exterior illumination of the plant. CO_2eq_ emissions were calculated according to Eq. (1)^30^

1 $${CO}_{2 eq, elect}={C}_{elect}\times {EF}_{elect}$$

where, CO_2 eq, elect_ is the GHG emissions associated with electricity consumption (kgCO_2 eq_ year^−1^); C_elect_ is the quantity of electricity consumed on the STP in a year (MWh/year) (obtained from questionnaire); EF_elect_ is the annual average of CO_2eq_ emission factor for the electricity sources (gCO_2 eq_ kWh^−1^). The percent contribution from thermal (coal and gas), nuclear, hydropower and renewable sources for the state of Himachal Pradesh was considered as per Table 1^35^. Accordingly, emission factors of 23 g CO_2_/KWh for hydropower, 820 g CO_2_/KWh for coal, 490 g CO_2_/KWh for gas and 12 g CO_2_/KWh for nuclear sources and 0 g CO_2_/KWh for renewable resources^36^ were used in the study.

**Table 1:** Sector wise contribution of various sources of power in Himachal Pradesh.

	Thermal		Nuclear	Hydropower	Renewable sources
	Coal	Gas			
% contribution	4.4%	1.6%	0.7%	71.8%	21.5%

### Emissions from activated sludge process

These emissions were calculated according to modified methodology^31,37^ as per Eqs. (2, 3, 4)

2 $${CO}_{2eq}^{ASP}={Y}_{{CO}_{2}}^{ASP}\times {O}^{ASP}$$

where: ${CO}_{2eq}^{ASP}$ is the total GHG emission, for activated sludge process (CO_2 eq_/day); ${Y}_{{CO}_{2}}^{ASP}$ is the production factor of CO_2_ in the aerobic process with activated sludge (= 1.37 kg CO_2_/kg BOD_5_)^30^; *O*^*ASP*^ is the amount of O_2_ needed for the process with activated sludge (kg O_2_/day)

3 $${O}^{ASP}={Q}_{ww\,inf}\times \left({BOD}_{5\,inf}-{BOD}_{5\,eff}\right)-1.42\times X$$

where: ${Q}_{ww inf}$ is the average daily flow (m^3^ day^−1^) (data was obtained from the questionnaire); ${BOD}_{5 inf}$ is the influent BOD_5_ (mg l^−1^); ${BOD}_{5 eff}$ is the effluent BOD_5_ (mg l^−1^).

For BOD estimation, 5-day BOD test as per 5210B method^38^ was used. Data for influent and BOD was procured as secondary data from STPs. Effluent samples were collected from the outlet of selected STPs biannually for 15 days for 2 years i.e. 2020 and 2021. The pH of wastewater samples was adjusted in the range 6.5–7.5. Water sample (152 ml) was taken in BOD bottle and 5–6 drops of nitrification indicator inhibitor was added and stirred properly. Gasket was kept in BOD bottle, and 3–4 drops of KOH solution was added and sensors were attached to the BOD bottle by using BOD system oxi-direct (Aqualytic make). Then BOD bottles were loaded in the system and kept in the incubator for five days at 20 °C. BOD readings were recorded after five days and expressed as mg l^-1^. An average of all values was obtained and used for the above calculations.

X is the biomass production, (kg day^−1^)

$$X=({Y}_{obs}\times {Q}_{wwinf}\times ({BOD}_{5inf}-{BOD}_{5eff}))/1000$$

${Y}_{obs}$ is the observed biomass yield, g volatile suspended solids (VSS) g^−1^ BOD_5_

4 $${Y}_{obs}=\frac{Y}{1+ {k}_{d}\times {\theta }_{c}}$$

where, *Y* is the biomass yield, 0.5 mg VSS mg^−1^ BOD_5_^30^; ${k}_{d}$ is the degradation rate of BOD_5_, 0.06 day^−1^^30^; θ_c_ is the sludge retention time for the activated sludge process. An average value of 10 days was used as obtained from the questionnaires.

### GHG emissions from secondary sludge

The secondary sludge emits CH_4_ directly and N_2_O indirectly. CH_4_ emission potential was calculated using Eqs. (5, 6, 7)^39^

5 $${CH}_{4\,emission}=\{\left[\left(U\times T\times EF\right)\right]\left(TOW-S\right)-R\}\times 28$$

where, CH_4 emissions_ is the CH_4_ emissions, (kg CO_2 eq_ year−^1^). TOW is the total organically degradable material in sewage (kg BOD year^−1^). S is the organic component removed as sludge (kg BOD year^−1^) (obtained from the questionnaire for each site and provided in the supplementary data). U is the fraction of population in the income group in the sampled area (0.23 for study area^39^). T is the degree of utilisation of treatment/discharge pathway or system (0.14 for study area^39^). EF is the emission factor (kg CH_4_ kg^−1^ BOD). R is the amount of CH_4_ recovered (kg CH_4_ year^−1^). This value was regarded as being zero because there no CH_4_ is recovered or flared in Himachal Pradesh.

Further, Emission factor^39^ is calculated as Eq. (6)

6 $$EF={B}_{o}\times MCF$$

where, B_o_ is the maximum CH_4_ producing capacity (0.6 kg CH_4_ kg^−1^ BOD)^40^. MCF is the methane correction factor (0.8, for study area^39^). Total organically degradable material in sewage (TOW) was calculated as Eq. (7)^40^.

7 $$TOW=P\times BOD\times 0.001\times I\times 365$$

where, P is the population. BOD is the region-specific per capita BOD (34 g person^−1^ day^−1^^40^). 0.001 is the conversion factor from grams BOD to kg BOD. I is the correction factor for additional industrial BOD discharged into sewers (considered 1.00 as default)^39^.

### Indirect nitrous oxide emissions

Indirect N_2_O emissions from the secondary sludge were calculated using Eqs. (8) and (9)^41^

8 $${N}_{2}{O}_{emission}= {N}_{effluent}\times {EF}_{effluent}\times \frac{44}{28}\times 265$$

where, N_2_O_emission_ is N_2_O emission, (kg CO_2 eq_ year^−1^). N_effluent_ is nitrogen in the effluent discharged to aquatic environments, (kg N year^−1^).

For N_effluent_ estimation, Effluent samples were collected from the outlet of selected STPs biannually for 15 days for 2 years i.e. 2020 and 2021. These samples were digested as per APHA Method 4500-N^42^ wherein samples were mixed with digestion reagent (K_2_SO_4_ + CuSO_4_ + H_2_SO_4_) and heated, to which sodium hydroxide-sodium thiosulphate solution was added and distilled into standard acid. The distillate was diluted and analyzed using digital photo colorimeter 312 with a red filter (630–660 nm). The absorbance was compared with calibration curve obtained using ammonia standards using ammonium chloride. An average of all values was obtained and used for the above calculations for each site.

EF_effluent_ is the emission factor for N_2_O emissions discharged to wastewater (kg N_2_O-N/kg N).

The default IPCC emission factor for N_2_O emissions from domestic wastewater nitrogen effluent is 0.005 kg N_2_O-N/kg N. This emission factor is based on limited field data and on specific assumptions regarding the occurrence of nitrification and denitrification in rivers and in estuaries. The first assumption is that all nitrogen is discharged with the effluent. The second assumption is that N_2_O production in rivers and estuaries is directly related to nitrification and denitrification and, thus, to the nitrogen that is discharged into the river.

The factor 44/28 is the conversion of kg N_2_O-N into kg N_2_O.

### GHG emissions from transportation and storage of the sludge

Since, the landfills for sludge storage were located at varying distances from the plants depending upon the availability of land for the same, transportation footprint was calculated using Eq. (10)^33^:

10 $${CO}_{2\,transportation}=Fuel\,consumption\times 2.6391$$

The data for fuel consumption was obtained from the questionnaire.

The off-site carbon dioxide equivalent emissions from landfilled sludge were calculated as per Eq. (11)^43^:

11 $${CO}_{2\,eq\,landfill}={Q}_{dry\,sludge}\times A\times B \times C \times D\times 25\times 16/12$$

where, *Q *_*dry sludge*_ is the dry weight of sludge produced annually, calculated on the basis that moisture content of sludge in the state is 65%^44^ (t/year); A is the methane conversion factor; 0.6^40^; for unmanaged shallow solid waste disposal sites (solid waste disposal sites that do not meet the criteria of managed solid waste disposal sites, which have depths of less than 5 m as per experts’ judgement). B is the degradable organic content of sludge. The IPCC default value of 0.5 was used for domestic sludge. C is the fraction of degradable organic content dissimilated to biogas. The IPCC default value of 0.5 (fraction) was used.

D is the Fraction of methane in the gas, for which the IPCC default value of 0.5 (fraction) was used. 16/12 is the conversion factor from C to CH_4_.

Values obtained from all these contributors to GHG emissions were then added to obtain the total emissions in the state.

## Results and discussions

Off-site greenhouse emissions are presented in Table 2.

**Table 2:** Greenhouse gas emission (tCO_2_) based on energy consumption of STPs in Himachal Pradesh.

STPs category	Energy consumption per plant (kWh year^−1^)	Volume of sewage treated per plant (m^3^ year^−1^)	GHG emissions (tCO_2 eq_ year^−1^)	
			Per plant	Total emissions in HP
< 1 MLD	56,666	1,73,333	199	4780
1—3 MLD	76,190	2,08,000	356	9270
> 3 MLD	1,08,571	4,07,333	752	6773
Total	–	–	–	20,823
C.D._0.05_	–	–	239	–

It is evident from the data presented in Table 2 that the energy consumption varied with the quantity of sewage treated and ranged from 56,666 KWh year^−1^ (< 1 MLD) to 1,08,571 kWh year^−1^ (> 3 MLD). The quantity of sewage treated also varied with the capacity from 1,73,333 m^3^ year^−1^ (< 1 MLD) to 4,07,333 m^3^ year^−1^ (> 3 MLD). Based on the capacity of STPs and energy consumption the greenhouse gases emissions also varied significantly. Higher GHG emission of 752 tCO_2 eq_ year^−1^ plant^−1^ was found with STPs of > 3 MLD capacity which was followed by plants of capacity between 1 and 3 MLD and < 1 MLD with emissions of 356 and 752 tCO_2 eq_ year^−1^ plant^−1^, respectively. Emissions from STPs of 1–3 and < 1 MLD capacities were found to be at par with each other, which could be due to the inconsistency in the amount of sewage received at these facilities.

Significantly higher amount of sewage being treated in larger plants requires long hours of operation as it was observed that at larger STPs aerators were running for around 8 h whereas, for smaller STPs they were running for as low as 2–3 h on an average every day at some facilities. Also, the pumps responsible for pushing the sewage and sludge from one tank to another at smaller facilities took lesser time in comparison to those treating higher volumes. Considerably higher GHG emissions may be due to larger volume of sewage treated and higher power consumption in > 3MLD STPs. Studies accounting for energy consumption in wastewater treatment infrastructures also pointed out that as the capacity of plants increased so did their energy consumption, suggesting thereby that the use of renewable energy sources in wastewater treatment may be helpful in reducing the footprints of STPs^45^. Studies by various researchers have reported a decrease in GHG emissions that result from electricity consumption during wastewater treatment because they used bio-generated electricity^46^.

**Figure 2:**
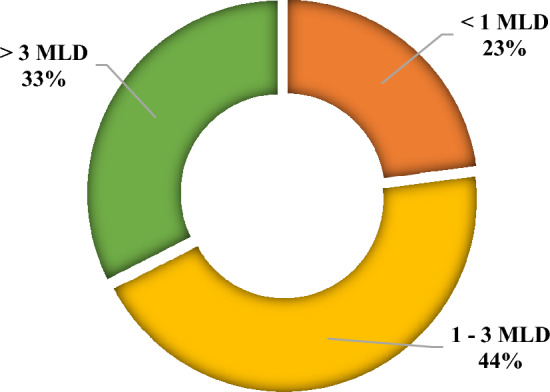
GHG emission potential of STPs based on energy consumption in Himachal Pradesh.

It is also evident from Table 2 that all STPs of the state emit 20,823 tCO_2 eq_ year^−1^ based on energy consumption with respective proportion of 9270 tCO_2_ (44%) came from 1 to 3 MLD STPs, followed by 6773 tCO_2_ (33%) that was contributed by > 3 MLD STPs. < 1 MLD STPs led to 4780 tCO_2 eq_ (23%) (Fig. 2). Though the emissions from a single plant are higher in case of plants of larger capacity, a greater amount of emission is seen from plants with 1–3 MLD capacity as their number is more than twice as compared to the larger plants in the state.

The results for on-site GHG emissions from activated sludge process at STPs are presented in Table 3

**Table 3:** Emissions of CO_2_, CH_4_ and N_2_O from activated sludge process in different STPs of Himachal Pradesh.

STP category	CO_2 eq_ per plant (tCO_2eq_ year^−1^)	CO_2 eq eff_ CH_4_ per plant (t CO_2 eq_ year^−1^)	CO_2 eq eff_ N_2_O per plant (t CO_2 eq_ year^−1^)	Total GHG emissions (t CO_2 eq_ year^−1^)
< 1 MLD	17.3	42.4	134.4	4,662
1–3 MLD	25.5	39.2	124.1	4,910
> 3 MLD	160.5	118.5	316.8	5,363
Total	–	**–**	**–**	14,934
C.D._0.05_	66.3	54.2	118.2	–

Activated sludge process is the most extensively used sewage treatment method in the state. As seen in Table 3 carbon dioxide emissions from activated sludge process varied significantly with plants of different capacities. Higher emission of 160.5 tCO_2 eq_ year^−1^ plant^−1^ was found from STPs of > 3 MLD capacity which was followed by plants of capacity between 1 and 3 MLD and < 1 MLD with emissions of 25.52 and 17.36 tCO_2 eq_ year^−1^ plant^−1^, respectively, which were at par with each other.

Methane emissions equivalent to 118.5 tCO_2_ were seen from > 3 MLD STPs, followed by 42.4 tCO_2_ from < 1 MLD. Further, it was followed by emissions from plants with capacity 1–3 MLD (39.2 tCO_2_) which were at par with each other. The anaerobic digestion in the primary sedimentation process and the whole sludge line could be a potential source of methane as also reported by some studies^26^.

Nitrous oxide emissions amounting to 316.8 tCO_2 eq_ were seen from > 3 MLD plants which were significantly higher than the emissions from < 1 MLD STPs seeing an emission of 134.4 tCO_2_ equivalent. It was at par with the emissions from STPs of capacity 1–3 MLD (124.1 tCO_2_ eq). Higher carbon dioxide equivalents of nitrous oxide emissions resulting from the activated sludge process were seen as compared to carbon dioxide and methane which could be attributed to a higher global warming potential of the gas amounting to as much as 280 times in comparison to CO_2_^45^. Additionally, these emissions could also be contributed from the rudimentary nitrification denitrification processes in the ASP^11^. A higher sewage holding time at the secondary tanks due to mismanagement at smaller STPs as compared to the STPs of capacity 1–3 MLD could attribute to the fact that the STPs of capacity 1–3 MLD and < 1 MLD were noticed to be at par with each other for various emissions from activated sludge process.

**Figure 3:**
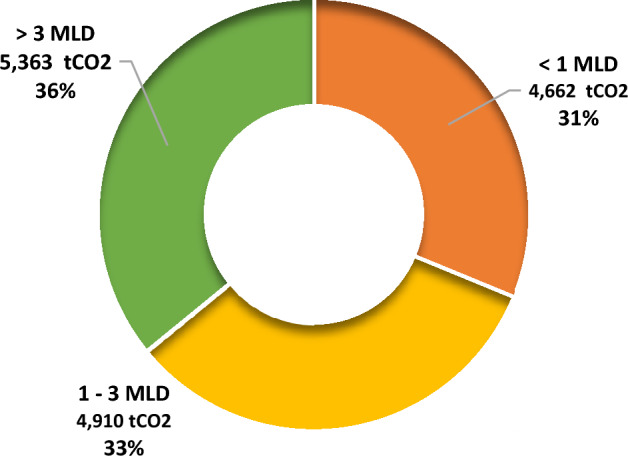
GHG emission potential of STPs based on activated sludge process in Himachal Pradesh.

As shown in Table 3, the activated sludge process of STPs in the state led to an emission of 14,934 tCO_2 eq_ wherein a respective proportion of 36% (5363 tCO_2_) came from > 3 MLD plants, followed by 33% (4910 tCO_2_) from 1 to 3 MLD STPs and 31% (4662 tCO_2_) which came from < 1 MLD STPs (Fig. 3). Though the number of larger STPs in the state is much lower than the others, still a higher contribution to emissions due to activated sludge process comes from them. Activated sludge process has been identified as one of the most greenhouse gas emitting processes among other technologies by various studies^47,48^. Usage of better and sophisticated technologies like MBR could solve the problem of emissions due to exceeded holding time in tanks due to unskilled and scarce manpower in the plants. The membrane filters could ensure that the microbes responsible for emissions are not present in the stagnant tanks.

Fuel consumption and GHG emissions due to transportation of sludge at different STP categories are presented in Table 4.

**Table 4:** GHG emissions from transportation of the sludge of different capacity STPs in Himachal Pradesh.

STP category	Fuel consumption per plant (l year^−1^)	GHG emissions (tCO_2 eq_ year^−1^)	
		Per plant	Total emissions in HP
< 1 MLD	6935.0	18.3	439
1–3 MLD	6772.7	17.9	465
> 3 MLD	9119.2	24.0	217
Total	–	–	1121
C.D._0.05_	NS	NS	–

Data presented in Table 4 suggests that the annual fuel consumption per plant varied from 6935 l (< 1 MLD) to 9119.2 l (> 3 MLD). Greenhouse gas emissions due to transportation of sludge to landfill sites was at par with each other for STPs of different capacities in the state as seen in Table 4. Larger distances of landfills from smaller plants as opposed to that from plants of higher capacity counteracted by the fact that larger plants produced a higher amount of sewage in comparison to their counterparts may have played a role in the above results. Similar amount of sewage sludge produced as seen in Table 5 at STPs of different capacities could be responsible for the non-significant result of these parameters. Emissions equivalent to 24 tCO_2_ from > 3 MLD STPs, 17.9 tCO_2_ from 1 to 3 MLD and 18.3 tCO_2_ from < 1 MLD were observed. Transportation of sewage sludge has been reported to be a contributor to GHG emissions and was dependant on the location of landfills with respect to the plants.^37,47^

**Table 5:** GHG emissions from storage of the sludge of different capacity STPs in Himachal Pradesh.

STP category	Q sludge per plant (tonnes year^−1^)	GHG emissions (tCO_2 eq_ year^−1^)	
		Per plant	Total emissions in HP
< 1 MLD	193.1	169.0	4056
1–3 MLD	178.3	156.0	4056
> 3 MLD	412.3	360.7	3247
Total	–	–	11,359
C.D._0.05_	168.9	147.7	**–**

**Figure 4:**
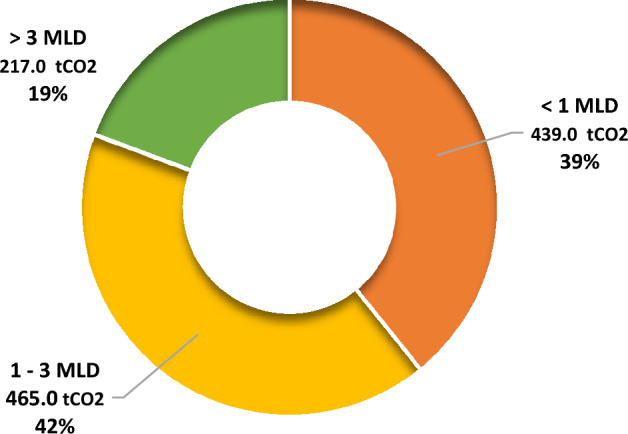
GHG emission potential of STPs based on transportation in Himachal Pradesh.

As seen in Table 4, the total GHG emissions from transportation of sludge of STPs in the state was observed to be 1121 tCO_2 eq_ per year, out of which 42% proportion (465 tCO_2 eq_) was contributed by 1–3 MLD STPs, 39% (439.0 tCO_2 eq_) by < 1 MLD STPs and 19% (217.0 tCO_2 eq_) by > 3 MLD STPs (Fig. 4). The difference in these emissions was due to a higher number of 1–3 MLD capacity STPs in comparison to others.

Quantity of sludge produced and GHG emissions from sludge storage are presented in Table 5.

As evident from Table 5, amount of sludge from STPs of different capacities varied from 178.3 t (1–3 MLD) to 412.3 t (> 3 MLD) annually. Greenhouse gas emissions due to storage of sludge in landfill sites was significantly higher for STPs of > 3 MLD than the other two STP categories which were at par with each other. Emissions equivalent to 360.7 tCO_2_, 156 tCO_2_ and 169 tCO_2_ were observed from > 3 MLD, 1–3 MLD and < 1 MLD STPs, respectively. The random distribution of STPs in the state due to its physiography leads to an uneven inflow to sewage to different plants. Consequently, the sludge produced per plant at < 1 MLD and 1–3 MLD were statistically at par with each other and were seen to have similar levels. The GHG emissions were distributed in accordance with the amount of sludge produced from different STPs. A direct relation between the capacity of wastewater treatment plants and the amount of sludge produced has been otherwise seen in studies^37^.

**Figure 5:**
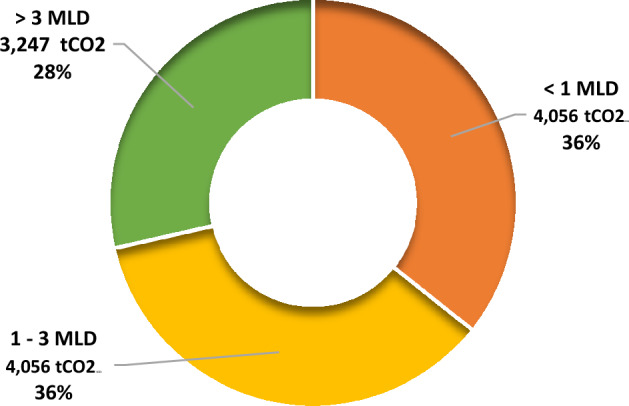
GHG emission potential of STPs based on storage of sludge in landfills in Himachal Pradesh.

Additionally, as seen in Table 5, total GHG emissions of 11,359 tCO_2 eq_ year^−1^ was observed from storage of sludge in landfill sites of STPs, out of which 4056 tCO_2 eq_ year^−1^ was from 1 to 3 MLD, 4056 tCO_2 eq_ year^−1^ was from < 1 MLD and 3247 tCO_2 eq_ year^−1^ was due to > 3 MLD STPs which was 36, 36 and 28% respectively (Fig. 5). Though the emissions from a single plant are higher in case of plants of larger capacity, a greater amount of emission is seen from plants with 1–3 MLD capacity and < 1 MLD capacity as they are more in number as compared to the larger plants in the state.

Total GHG emissions from STPs of different categories in the state is given in Table 6.

**Table 6:** GHG emissions from STPs of different capacities in Himachal Pradesh.

STP category	GHG emissions (tCO_2 eq_ year^−1^)	
	Per plant	Total emissions in HP
< 1 MLD	581	13,937
1–3 MLD	719	18,700
> 3 MLD	1733	15,599
Total	–	48,237

As evident from Table 6, GHG emissions ranged from 581 to 1733 tCO_2 eq_ annually from STPs of different capacities in the state. Emissions equivalent to 581 tCO_2_, 719 tCO_2_ and 1733 tCO_2_ per plant were observed from < 1 MLD, 1–3 MLD and > 3 MLD STPs, respectively, in the state. Additionally, total emissions of 13,937, 18,700 and 15,599 tCO_2_ were seen from plants of capacity < 1 MLD, 1–3 MLD and > 3 MLD STPs, respectively. The STPs in Himachal Pradesh contributed to 48,237 tCO_2 eq_ emissions of greenhouse gases per year with a total sewage treatment capacity of 99 MLD which is equivalent to 1.3 kg CO_2 eq_/m^3^ of sewage treated. It was concluded that this emission was considerably higher when compared to wastewater treatment plants of similar capacity in Mumbai metropolitan region, where total GHG emission of 0.22 kg CO_2 eq_/m^3^ were recorded by researchers^49^. Studies have also revealed that with upgradation in wastewater treatment technologies, energy recovery through biogas and proper maintenance of STPs would lead to a reduced footprint despite of the increased inflow of wastewater into the plant.

**Figure 6:**
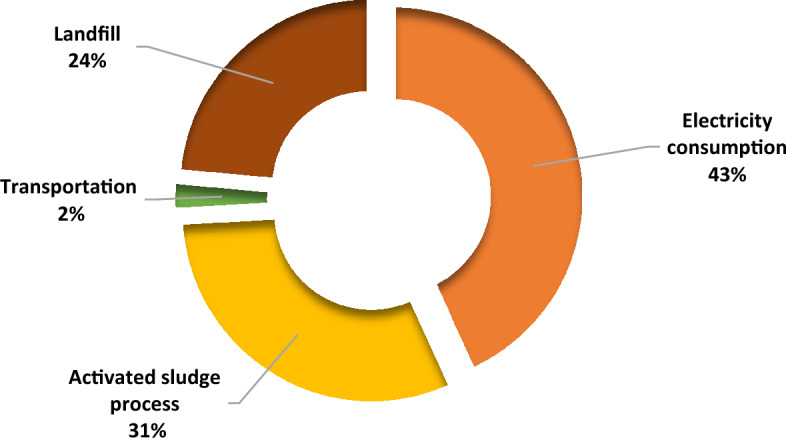
Total GHG emissions from various sources in STPs in Himachal Pradesh.

Total emissions from various sources in STPs in Himachal Pradesh is summarised in Fig. 6.

The major contribution resulted from electricity consumption (20,823 tCO_2 eq_) which accounted for 43% of the total emission. Long hours of operation of STPs in the state using time consuming processes like activated sludge process could be responsible for these emissions. Use of renewable sources of energy harvested at the site of operation could be used to reduce these emissions. It was followed by activated sludge process where 14,934 tCO_2 eq_ were released, forming around 31% of the emissions. ASP was also identified as one of the major sources of GHG emissions by some researchers^11,22,50^ Landfills were the next contributors with 24% share with emission amounting to 11,359 tCO_2 eq_ followed by transportation which led to 2% of emissions (1121 tCO_2 eq_) (Fig. 6). Transportation has been reported to be a contributor to negligible amount of emissions in comparison to other sectors^22,37^ in facilities where high energy consuming processes like ASP were in use.

The GHG emission analysis revealed that STPs in Himachal Pradesh have a small impact at national level, corresponding to 0.005% of total CO_2 eq_ emissions in the state. Nevertheless, the optimization of these infrastructures can be significant at a local scale and help improve the footprint of these areas.

## Conclusion

The findings of the study demonstrate that STPs in Himachal Pradesh contribute a significant annual total of 48,237 tCO_2_ eq GHG emissions. The results also highlight that the primary source of emissions is energy consumption during STP operations, emphasizing the necessity of replacing non-renewable energy sources with renewable ones. Additionally, the activated sludge process, storage of sludge, and transportation were identified as sources of STP emissions and need further examination to achieve a zero-emission scenario. The study recommends a careful plant design and optimized operation of the activated sludge process, along with the use of renewable energy sources like solar energy, as potential measures for reducing the environmental footprint of STPs and making them more sustainable. These recommendations provide valuable insights for STP management to mitigate GHG emissions and promote sustainable practices in the field of wastewater management.

## Supplementary Information

Supplementary Information.

## Data Availability

The datasets used and/or analysed during the current study available from the corresponding author on reasonable request.
